# Effects of a single 20 mg dose of letrozole on ovarian function post dominant follicle selection: an exploratory randomized controlled trial

**DOI:** 10.1186/s13048-017-0303-x

**Published:** 2017-01-21

**Authors:** H.C.M. Allaway, D.R. Chizen, G.P. Adams, R.A. Pierson

**Affiliations:** 1 Department of Obstetrics, Gynecology & Reproductive Sciences, College of Medicine, University of Saskatchewan, 103 Hospital Drive, Saskatoon, SK S7N 0 W8 Canada; 2 Department of Veterinary Biomedical Sciences, Western College of Veterinary Medicine, University of Saskatchewan, Saskatoon, SK Canada; 3 Present Address: Department of Kinesiology, Pennsylvania State University, State College, PA USA

**Keywords:** Aromatase inhibitor, Follicular phase, Ovulation, Estradiol, Endometrium

## Abstract

**Background:**

Our objective was to explore the impact of a single dose of an aromatase inhibitor (letrozole) administered at defined times of the follicular phase or immediately after ovulation on dominant follicle development, luteogenesis and new follicle wave emergence.

**Methods:**

A prospective pilot study using a randomized complete block, controlled, open label design was conducted at an academic clinical research center. Forty-five healthy, female volunteers (25.5 ± 0.9 years, BMI 25.0 ± 0.6 kg/m^2^) who had not taken hormonal contraceptives for a minimum of 2 months were recruited. A 20 mg dose of Letrozole was administered once orally in each of 3 groups when the dominant follicle reached a diameter of 1) 12 mm, 2) 18 mm, 3) the first day following ovulation (post-ovulation), or 4) treatment was withheld (control). Serial ultrasonography and phlebotomy began on day 4 of the menstrual cycle and continued for 1.5 menstrual cycles. Participants recorded menses and daily events in a life events calendar for the duration of the study. Demographic and single point measurements were compared among groups by ANOVA. Changes in hormone concentrations over time were compared among groups by repeated measures ANOVA. Kruskal-Wallis tests were used for non-normally distributed data.

**Results:**

The dominant follicle in all treatment groups ovulated. There were no differences among experimental groups in peak follicle diameter, follicular growth rate, endometrial thickness at ovulation or inter-ovulatory interval. Plasma concentrations of estradiol dropped, while FSH and LH concentrations rose following treatment in all treatment groups. Plasma FSH and LH concentrations were higher in the 18 mm group compared to the 12 mm and post-ovulation groups (*P* < 0.02).

**Conclusion:**

Administration of a single 20 mg dose of Letrozole at the times of the menstrual cycle we examined did not induce dominant follicle regression or failure of corpus luteum formation. Letrozole-induced suppression of estradiol synthesis by the dominant follicle was not detrimental to follicle growth or ovulation following follicle selection, likely due to increased circulating concentrations of FSH and LH resulting from a lack of estradiol-induced suppression of the hypothalamic-pituitary-ovarian axis.

**Trials registration number:**

Clinical trials registration number NCT01046578.

## Background

The traditional model of folliculogenesis during the human menstrual cycle posits that a single cohort of antral follicles in the ovary begins to grow at the beginning of the menstrual cycle and a dominant follicle is physiologically selected in the mid-follicular phase: ovulation of the dominant follicle occurs approximately 2 weeks following the onset of menses [1]. However, a more current model has evolved through the use of serial transvaginal ultrasonography which enabled detailed characterization of follicular and luteal dynamics through the identification of definitive ovarian points of reference; i.e., the occurrence of ovulation and the emergence of follicular waves [2–4]. Menses occurs in the middle of the inter-ovulatory interval and the final of 2 or 3 waves of follicle development culminates in ovulation. Descriptions of antral follicle waves in women are consistent with follicular wave dynamics observed during the estrus cycles of several domestic animal species [5–8].

Data from Canada and the United States emphasize the need for a better understanding of the regulation of ovarian function [9–15], as the use of emergency and cyclic hormonal contraception increases. In 2006, approximately 450,000 pregnancies were documented in Canada and 20% ended in elective termination [10, 11]. Similarly, 51% of the 6.7 million documented pregnancies in the United States in 2008 were unintended [13, 14], with 40% of unintended pregnancies ending in elective termination (20% of all pregnancies) [14]. The Centers for Disease Control reported 11% of 15–44 year olds and 23% of 20–24 year olds had ever used an emergency contraceptive [12]. The reasons for use were fear of contraceptive failure (45%) and unprotected sex (49%) [12]. Croxatto et al. [16, 17] have demonstrated the occurrence of ovulation following emergency contraception administration varied depending on the size of the follicle at administration. The closer the follicle size was to preovulatory, the less likely treatment with a full or half dose of emergency contraception was going to prevent ovulation [16, 17]. Thus, the mechanism of action for combined hormonal contraception use as an emergency contraceptive is not well understood and investigations into the removal of endogenous estrogen would provide a unique opportunity to evaluate mechanisms of steroidogenesis impact on folliculogenesis.

The non-steroidal aromatase inhibitor (AI) Letrozole (Femara^TM^) reversibly inactivates the aromatase enzyme [18–20] and prevents the enzymatic conversion of androgens to estrogens [19, 21]. Letrozole is the most commonly used AI for ovulation induction and has been used successfully when administered between days 3 and 7 of the follicular phase as small 5-day dose (2.5 or 5.0 mg/day) and large single dose (20 mg) regimens [18, 19, 22–24]. To date, there are no reports of AI use initiated at other points of follicle growth in humans.

The decline in FSH coupled with the rise in estradiol (E_2_) early in the follicular phase is well documented; however, the response of post-deviation follicles to decreasing E_2_ and increasing FSH is not well documented [25, 26]. In the bovine model of ovarian function, neither a single nor 3 day AI dosing protocol was able to induce regression of a dominant follicle nor cause an increase FSH release [27, 28]. Yapura et al. [27, 28] observed a lengthened follicular dominance and increased secretion of luteinizing hormone (LH) with a single and 3 day AI dosing protocol. Single and multi-day dosing in humans and animal models demonstrated similar results, therefore the use of the single day protocol should be viewed as most appropriate [22–24, 27, 28]. A better understanding of the role of estrogen in controlling folliculogenesis, and the effects of aromatase inhibitors on post-deviation ovarian function may lead to the development of better protocols for both fertility and contraception. Thus, the overall objectives of the present study were to evaluate mechanistically the ovarian and endocrine effects of Letrozole treatment when administered at the time of dominant follicle selection, the immediate pre-ovulatory period and immediate post-ovulatory period during natural menstrual cycles.

## Methods

A prospective pilot study was conducted using a controlled, randomized complete block, open label design at a single center.

### Participants

Forty-five women between the ages of 18 and 35 years (25.5 years ± 0.9; mean ± SD) with a body mass index (BMI) of 18–32 (25.0 ± 0.6) were enrolled and 41 women completed the study. Informed consent was obtained from all women prior to the initiation of study procedures. All participants were self-reported non-smoking, healthy women with regular menstrual cycles with no contraindication to contraceptive or aromatase inhibitor use (as determined by medical history and physical examination). The women were naïve to the use of hormonal contraceptive or were required to discontinue use of hormonal contraceptives ≥ 2 months before the study. Participants were asked to abstain from intercourse during the study; however, participants were also provided with barrier contraceptives (condoms) throughout the study to prevent pregnancy if they were requested.

### Treatments

At the time of the first ultrasound examination, women were assigned to 1 of 4 experimental groups in a randomized, complete block design using a random number generator by HCMA. Women in the respective groups were given a single 20 mg oral dose of letrozole (Femara, Novartis Pharmaceuticals Canada, Inc., Dorval, Quebec) when the largest ovarian follicle after menses was first detected at a diameter of 1) 12 mm (*n* = 10; peri-selection group), 2) 18 mm (*n* = 10; pre-ovulatory group), or 3) within 24 h after ovulation (*n* = 10; post-ovulation group), or 4) were given no treatment (*n* = 11; control group).

### Ultrasonography

**Fig. 1:**
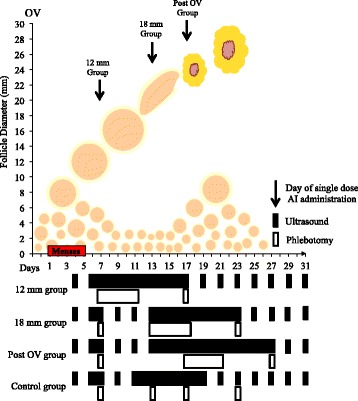
Schematic diagram of the study protocol

Ovarian follicular and endometrial development were evaluated by transvaginal ultrasonography using a 6–9 MHz convex-array transducer (Ultrasonix RP, Vancouver, BC, Canada). Most examinations (95%) were performed by one operator (HCMA); the remaining examinations were performed by co-authors. Participants were monitored for 1.5 menstrual cycles (i.e., from the first menses of the observational period to the first ovulation after the second menses). Beginning on day 4 (day 1 = first day of menses), ultrasonographic examinations were performed every-other-day until the day of letrozole treatment in the peri-selection (12 mm) group or until the dominant follicle was ≥ 16 mm in diameter in all other experimental groups when ultrasound examinations were done daily thereafter for a minimum of 11 days or until the fate of the dominant follicle was determined (i.e., regression, formation of an anovulatory follicular cyst, hemorrhagic anovulatory follicle (HAF), luteinized unruptured follicle (LUF) or ovulation (OV)). Following ovulation, women in the control group were then examined every-other-day until the post-treatment ovulatory follicle attained a 16 mm diameter. Daily examinations then resumed until the subsequent ovulation. In all treatment groups, subsequent ultrasound examinations were conducted every-other-day from the time that the dominant follicle’s fate was determined until the post-treatment ovulatory follicle was ≥ 16 mm in diameter; daily examinations then resumed until the subsequent ovulation. A schematic representation of the study protocol is shown in Fig. 1.

The number and diameter of antral ovarian follicles ≥ 2 mm were imaged and tabulated during each ultrasound examination to assess the day of wave emergence. The average of two orthogonal measurements was used as the mean follicle diameter for all follicles > 10 mm. The dominant follicle was defined as the follicle ≥ 10 mm that surpassed the diameter of all other follicles in the cohort by ≥ 2 mm in diameter [2, 3]. Subordinate follicles were defined as all other follicles in the cohort with the dominant follicle and the 1^st^ subordinate follicle was the largest of the subordinate follicles. Co-dominant follicles were defined as two follicles reaching > 13 mm [29]. Ovulation was defined as the disappearance of a follicle ≥ 12 mm in diameter detected ultrasonographically the previous day, followed by subsequent visualization of a corpus luteum [30]. Wave emergence was defined retrospectively as the day when the largest follicle of each wave was first identified at 4–5 mm. The identity and non-identity methods were used to characterize changes in follicle diameter during the study [2, 7, 31]. The diameter profiles of individual follicles that grew ≥ 8 mm over time for each woman were tabulated using the day of follicular wave emergence as the point of reference [2].

Endometrial thickness and pattern also were recorded during each ultrasound examination following standard operating procedures of the WHIRL, as previously described [32]. Briefly, endometrial thickness was measured as the distance from the anterior stratum basalis-myometrial junction to the posterior stratum basalis-myometrial junction in the mid-sagittal plane. The transverse and sagittal planes of section which represented the largest dimensions of the fundal aspect of the endometrium were used for all measurements. Endometrial echotexture pattern was assessed each day as either an M, A, B, C or D pattern.

### Blood sampling

In the treatment groups blood samples were taken daily for 5 days beginning on the day of treatment. The interval was defined as the observation interval. If the fate of the extant dominant follicle was not determined after 5 days, blood samples were taken every-other-day until the dominant follicle met one of the defined fates. In all participants a single blood sample was taken 5–9 days after the fate of the follicle was known. A final blood sample was taken when the pre-ovulatory follicle of the second cycle reached 18 mm. In the control group, blood samples were taken at times equivalent to the day of treatment in the other groups (i.e., 12 mm follicle, 18 mm follicle, and 24 h post-ovulation), 5–9 days following ovulation and when the pre-ovulatory follicle reached 18 mm. Blood samples were collected in 7 mL tubes and allowed to coagulate for 30–45 min at room temperature before centrifugation for 30 min at 700 × g. Serum samples were stored at −20 °C until all participants completed the study.

### Daily events charts

All volunteers were provided with an event diary to record menstrual patterns, concomitant medications and any adverse events that occurred during the study.

### Hormone assays

Competitive fluorescence immunoassays (Immulite^TM^, Siemens Healthcare Diagnostics Inc., Tarrytown, NY, USA) were used to measure serum FSH and LH concentrations and validated radioimmunoassays were used to measure serum E_2_ and progesterone (P_4_) concentrations. All assays were performed in a single batch and conducted at Prairie Diagnostic Services at the University of Saskatchewan. Minimum detectible limits were: FSH: 0.1 mIU/mL; LH: 0.1 mIU/mL; E_2_: 1.4 pg/mL; and P_4_: 0.02 ng/mL. Intra-assay coefficients of variation for low, medium and high reference concentrations, respectively, were 3.0, 2.9 and 1.6% for FSH, 2.6, 2.4 and 1.6% for LH, and 3.9, 6.3 and 7.6% for P_4_. Intra-assay coefficients of variation for low and high reference concentrations, respectively, were 2.4 and 10.9% for E_2_. Inter-assay coefficients of variation were 4.7 and 13.2% (low, high) for E_2_ and 3.9, 6.3 and 7.6 (low, medium, high) for P_4_. The magnitude of the decrease in plasma estradiol concentration following treatment was calculated using the equation ([E_2_ day of treatment] – [E_2_ day of nadir])/(day of nadir – day 1).

### Statistical analyses

Sample size was determined by the mechanistic and pilot study nature of the trial. All analyses were based on intent-to-treat. Statistical analyses were performed using SAS version 9.2 (SAS Institute, Cary, NC, USA). Single-point measurements were compared among groups by analysis of variance. Changes in hormone concentrations (E_2_, FSH and LH) and endometrial thickness over time were compared among groups by analysis of variance for repeated measures (PROC MIXED). Endometrial patterns and intervals from treatment to ovulation were compared using Kruskal-Wallis tests. Significance was set at *P* < 0.05. Results are expressed as the mean ± SEM.

## Results

**Fig. 2:**
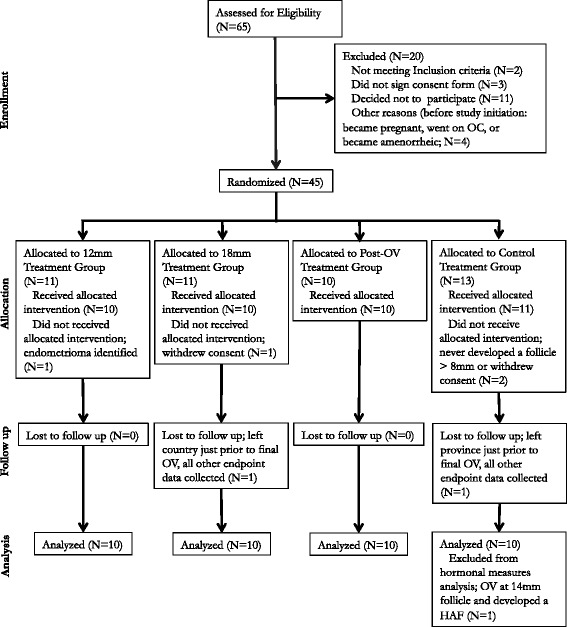
Flow of participants through the study from enrollment to data analysis

The flow of participants through the study protocol is shown (Fig. 2). Participants who had aberrations in luteal follicle wave dynamics were followed ultrasonographically over a longer time and had extra days of blood sampling (data not shown). The age and BMI of participants were similar among treatment groups (Table 1; P > 0.4). No adverse events were observed in our subjects. Forty-one participants completed the pilot study. Four participants were excluded or withdrew from the study prior to receiving treatment and were not included in the analyses. One participant in the 18 mm group and one in the control group were lost to follow-up just prior to ovulation at the end of the study and is included in all analyses. One participant in the control group ovulated at a small follicle diameter (>14 mm), which was not observed by ultrasound within 24 h, thus this participant was excluded from analyses of hormone data.

**Table 1:** Descriptive Statistics for age and BMI (mean ± SEM) of participants

Experimental Group	*N*	Age (years)	BMI (kg/m^2^)
12 mm	10	24.8 ± 1.9	25.4 ± 0.8
18 mm	10	28.0 ± 1.6	24.5 ± 1.1
Post OV	10	24.0 ± 1.6	24.8 ± 1.4
Control	11	25.2 ± 1.8	25.4 ± 1.1
*P*-value		0.404	0.934

### Follicular and luteal dynamics

**Fig. 3:**
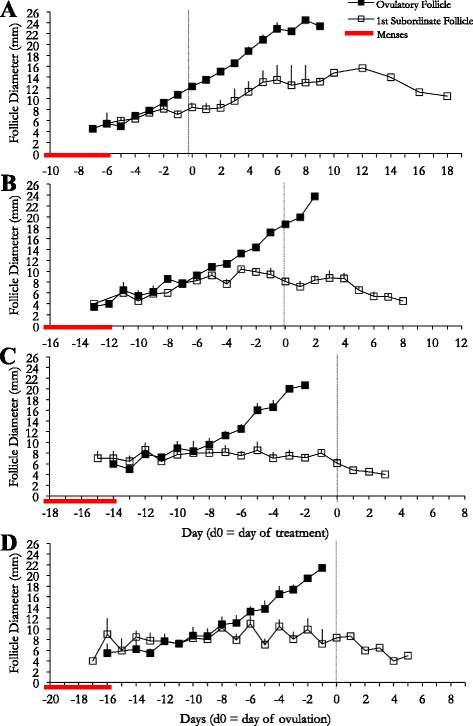
Mean follicle diameter profiles of the treatment groups. The 12 mm group (*n* = 10; **a**), 18 mm group (*n* = 10; **b**), post-OV group (*n* = 10; **c**), and control group (*n* = 11; **d**) are shown separately. Data are shown for 9 days following ovulation and are centralized to the day of treatment for each respective treatment group or day of OV in the control group

The mean diameter profiles of the dominant and first subordinate follicles of the ovulatory wave for each treatment group are shown (Fig. 3). The dominant follicles extant at the time of treatment in all study groups ovulated. Ovulation was confirmed by ultrasonographic observation of a corpus luteum and a subsequent rise in P_4_ on days 5–9 [33]. In the 12 mm group, 3 of 10 (30%) women developed co-dominant follicles: one follicle ovulated and the other regressed in one woman and both follicles ovulated in 2 women. One of the 10 (10%) women in the 18 mm group developed co-dominant follicles; one follicle ovulated and the other regressed. In the control group, one of the 11 women (9%) developed co-dominant follicles; one follicle ovulated at 13 mm and the other developed into a HAF. Data from this participant were excluded from hormonal analysis.

The interval from treatment to ovulation differed among groups (*P* < 0.0001; Table 2). Peak follicle diameter was greater in the 12 mm group than that of the 18 mm and control groups (*P* < 0.04). The interval from treatment to ovulation was longest in the 12 mm group and shortest in the 18 mm group. There were no differences among study groups in the rate of growth of the dominant follicle from treatment to ovulation, or to peak follicle diameter before follicle rupture was observed in the post-ovulation group. (P > 0.1; Table 2).

**Table 2:** Mean follicular end points (mean ± SEM) among experimental groups

Experimental Group	*N*	Day of treatment (d)	Peak diameter (mm)	Follicular growth rate (mm/d)	Interval from treatment to OV (d)
12 mm	10	9.2 ± 0.4	23.2 ± 1.0	1.5 ± 0.1	7.4 ± 0.6
18 mm	10	14.5 ± 1.1^a^	19.8 ± 0.6	1.3 ± 0.1	1.9 ± 0.2^a^
Post OV	10	17.9 ± 0.8^a, b^	20.7 ± 1.3	1.5 ± 0.1	−1.0 ± 0.0^a, b^
Control	11	NA	19.8 ± 1.4	1.2 ± 0.1	NA
*P*-value		0.0001	0.1266	0.1809	0.0001

All comparisons are within columns

^a^difference from 12 mm group (*P* < 0.05)

^b^difference from 18 mm group (*P* < 0.05)

The interval from detection of an 18 mm follicle to ovulation was shorter in the 18 mm treatment group than in the control group (41.8 ± 13.1 h vs. 72.5 ± 34.9 h, respectively; *P* = 0.006). The 12 mm, 18 mm, post ovulation and control groups had similar intervals from ovulation to new follicular wave emergence (0.4 ± 4.5 d, −0.2 ± 2.5 d, 0.5 ± 3.0 d, 0.8 ± 3.2 d, respectively; *P* = 0.925).

Day 5–9 serum P_4_ concentrations were 22.9 ± 5.5 ng/mL, 16.3 ± 1.8 ng/mL, 13.0 ± 1.4 ng/mL and 24.1 ± 5.7 ng/mL in the 12 mm, 18 mm, post-ovulation and control groups, respectively (*P* = 0.1899) and were above the clinically defined minimum P_4_ concentration required to confirm ovulation. No differences were observed among groups with regard to P_4_ concentrations, inter-menstrual interval and inter-ovulatory interval.

### Circulating hormone concentrations

**Fig. 4:**
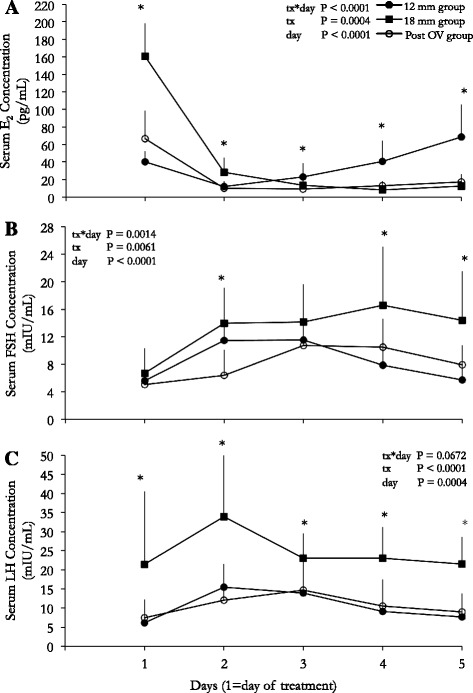
Change in estradiol, FSH and LH over the observation interval. **a** Mean changes in E_2_ concentration over the observation interval in the treatment groups (day 1 = day of treatment). **b** Mean changes in FSH concentration over the observation interval (day 1 = day of treatment) in the treatment groups. **c** Mean changes in LH concentration over the observation interval (day 1 = day of treatment) in the treatment groups. Differences among treatment groups are identified within days (*P* < 0.05). * Within day comparisons among experimental groups. *N* = 10 for 12 mm, 18 mm and post-OV groups

Changes in serum concentrations of E2, FSH and LH in the treatment groups are shown (Fig. 4). The pattern of change in serum E_2_ concentration across the observation interval differed among the 3 treatment groups (*P* < 0.0001; Fig. 4a). The 18 mm group exhibited higher (*P* < 0.0001) E_2_ concentrations on the day of treatment, which was attributed to the presence of a pre-ovulatory dominant follicle. Following treatment E_2_ concentrations decreased in all treatment groups (*P* = 0.0004). The 18 mm group E_2_ concentrations reached baseline on the third day after treatment and remained at baseline until the 5^th^ day (*P* = 0.8563). The 12 mm group E_2_ concentrations increased (*P* = 0.01) from 2 to day 5. The magnitude of the initial drop in E_2_ concentration in the 18 mm group was larger than in both the 12 mm and post ovulation groups (*P* = 0.04).

Plasma FSH concentrations increased and subsequently decreased to pretreatment concentrations over the 5-day observation interval in all treatment groups (*P* = 0.0014; Fig. 4b). The statistical interaction was attributed to the immediate FSH increase from day 1 to 2 in the 12 and 18 mm groups (*P* < 0.0001).

Following treatment, plasma LH concentrations in all treatment groups initially increased (*P* = 0.0013; Fig. 4c) and then decreased to pretreatment levels by the end of the observation interval. The mean plasma LH concentrations over the observation interval were higher in the 18 mm group versus the 12 mm and post ovulation groups (*P* < 0.02).

The maximum concentrations of FSH and LH following treatment differed among treatment groups (*P* < 0.03). The 18 mm group had higher FSH and LH concentrations than the 12 mm and post ovulation groups (*P* < 0.05); maximum FSH and LH concentrations did not differ between the 12 mm and post ovulation groups (P > 0.26).

### Endometrial dynamics

**Fig. 5:**
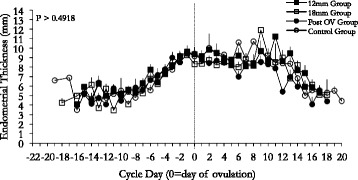
Mean profiles of endometrial thickness. Women in all treatment groups, 12 mm (*n* = 10), 18 mm (*n* = 10), 24 h post ovulation (*n* = 10), and control (*n* = 11) are identified. Data are shown for one inter-menstrual interval and are centralized to the day of ovulation. No significant effect of day was observed for endometrial thickness

Changes in mean endometrial thickness beginning at the time of pre-treatment menses are shown (Fig. 5). Mean cycle endometrial thickness and endometrial thickness and pattern at ovulation were not different among experimental groups (*P* = 0.1124). There were no differences in the interval from menses to ovulation, inter-ovulatory interval or inter-menstrual interval among experimental groups (*P* = 0.2; Table 3).

**Table 3:** Descriptive statistics for physiologically relevant intervals (mean ± SEM) among experimental groups

Experimental Group	*N*	Interval from menses to OV	Inter-menstrual interval	Inter-ovulatory interval	Interval from treatment to menses
12 mm	10	16.6 ± 0.5	27.6 ± 1.1	29.5 ± 1.6	19.7 ± 1.3
18 mm	10	16.4 ± 1.1	29.6 ± 1.4	26.9 ± 2.0	16.1 ± 0.6^a^
Post OV	10	16.9 ± 0.8	28.0 ± 1.0	23.9 ± 3.0	11.1 ± 0.9^a, b^
Control	11	17.2 ± 1.0	28.5 ± 1.1	29.0 ± 1.1	NA
*P*-value		0.9284	0.6551	0.2014	0.0001

All intervals are in days

*N* = 9 for 18 mm group and *N* = 10 for control group in inter-ovulatory interval column

^a^difference from 12 mm group (*P* < 0.05)

^b^difference from 18 mm group (*P* < 0.05)

## Discussion

The dominant follicle observed in all treatment groups ovulated and there were no differences among experimental groups for peak follicle diameter, follicular growth rate, nor endometrial thickness at ovulation. As expected E_2_ concentration declined, while both FSH and LH concentrations increased following administration of a single 20 mg dose of AI in all treatment groups. Differences among groups in P_4_ concentrations, inter-menstrual interval and inter-ovulatory interval were not observed, therefore, we concluded that there was no effect of treatment on luteogenesis.

Concentrations of serum E_2_, FSH and LH on the day before receiving treatment in each group did not exceed previously published ranges for the specified physiologic time points in our laboratory [3] nor did they differ from the control group at the same time point. Decreased E_2_ was observed within 24 h of AI administration in all women, supporting our hypotheses that treatment with an AI would cause a transient drop in E_2_. These findings contrast studies in the bovine model, where increasing plasma E_2_ concentrations were observed for 4 days following AI administration in metestrous. The E_2_ increase was associated with a simultaneous increase in follicle diameter [28], which is similar to the continued follicle development in all our treatment groups despite the drop in E_2_ concentrations. In the 12 mm group an increase in E_2_ concentrations began 2 days after AI administration and was attributed to the health and continued growth of the pre-ovulatory dominant follicle, while the absence of a rise in E_2_ in the 18 mm and post-ovulation groups has been attributed to the physiologic drop in E_2_ secretion following ovulation [3]. A compensatory mechanism for the preservation of follicle growth and ovulation following the acute drop in E_2_ after AI administration may be explained by the observation of increases in FSH and LH concentrations.

All treatment groups exhibited a rise in FSH following treatment in the present study. Garcia-Velasco et al. [34] demonstrated a 7 day delay in a rise in FSH when Letrozole was administered during the luteal phase. Both documented rises in luteal FSH concentrations in women contrast with a lack of FSH change in the bovine model [27, 28]. In the present study, all three groups exhibited a typical mid follicular phase rise in FSH during the 4 days immediately following treatment. A 4-day rise in FSH following aromatase inhibitor treatment later in the follicular phase (day 12 and 18) appeared to elongate the FSH rise thus potentially allowing multiple dominant follicles to develop.

Circulating LH concentrations reached maximum concentrations within 24 h of treatment and returned to initial concentrations by the end of the observation interval in all groups. The rise in LH concentrations within 24 h is consistent with reports of AI treatment in the bovine model [27, 28]. A sustained elevation in LH following cessation of follicular phase AI administration has not previously been reported in women [18, 20, 22–24]. Bedaiwy et al. [35] documented no difference in LH concentrations in AI treated cycles compared to natural cycles up to day 11 of the menstrual cycle; however, treatment with 2.5 mg AI led to significantly lower LH on the day of hCG administration. It has been suggested that the prolonged elevation in LH following aromatase inhibitor treatment in the follicular and luteal phases indirectly enhances dominant follicle development [27, 28].

When treatment was administered at a 12 mm follicle diameter the selected follicle continued to grow, and attained a larger pre-ovulatory size compared with the 18 mm treatment group and control group and there was a tendency for the follicle to be larger than observed in the post-ovulation group. However, in the 18 mm group there was no change in follicle diameter at ovulation compared to the control group. The trend towards a larger ovulatory follicle in the 12 mm group may be related to the acute drop in E_2_ in the mid-follicular phase. We postulated that the compensatory rise in FSH and LH following an AI induced drop in E_2_ accentuated follicular growth. However, within the constraints imposed by our study design, we were unable to conclude that AI treatment affects ovulatory follicle diameter.

Our observation of an unaltered growth profile is in contrast with the bovine model where a single, large dose of letrozole after follicle selection prolonged the interval from treatment to ovulation [27, 28]. To date, there has been no in depth evaluation of the effects of AI use on follicle growth profiles when administered prior to dominant follicle selection (i.e., 10 mm diameter). In retrospect, it would have been useful to have include a treatment group in which women received Letrozole on the 3^rd^ day following menses an prior to follicle selection. Initiation of AI treatment prior to selection has been documented in ovulation induction, which appears to release negative feedback on FSH secretion and stimulate further growth of the extant follicular wave; however, available studies did not measure changes in FSH during treatment [19, 20, 22, 24, 35]. In natural menstrual cycles, the duration of the FSH rise at wave emergence is short and it is presumed that this is due to the negative feedback system of E2 and inhibin B. This mechanism is postulated to ensure monovulation [20].

In the present study, multiple dominant follicles were developed by women in the 12 mm, 18 mm and control groups in the follicular phase of the treatment cycle. In the 12 mm group, 20% of the women ovulated two follicles during the treatment period, in contrast with the reported 4% natural occurrence of double ovulation [26, 29]. Another participant in the 12 mm group developed a second dominant follicle that regressed. This observation is conceptually similar to a recent study in the bovine model wherein the 1^st^ subordinate follicle of animals given an AI in the follicular growth phase (day 1–3) exhibited larger mean diameters [27]. It was postulated that an extended mid follicular phase FSH rise in women allowed a subordinate follicle to be rescued from atresia. In both the 18 mm and control groups, a second dominant follicle grew but either regressed (10%) or resulted in ovulatory failure (9%). Ovulatory failure occurs in approximately 5% of cycles of regularly cycling women [36] and 10% of cycles in infertile women [37, 38]. An increased incidence of ovulatory failure was not observed in the present study.

We were limited in our ability to evaluate the impact of AI treatment on luteal function. We hypothesized that AI treatment would result in ovulatory failure and hence did not anticipate a need for the evaluation of serial luteal phase P_4_. We were also limited in our ability to directly assess differences between the control group in this pilot study and each of the treatment groups across the entire treatment timeframe. Blood draws in the control group were timed only to coincide with the times of letrozole treatment. An optimal design to facilitate comparison of the control group to the treatment groups during the observation interval would have required volunteers to submit to daily blood collection for approximately 15 consecutive days. We were already limited by our ability to attract volunteers for the study protocol as executed. We were also financially unable to support a separate control group in this study for each treatment group.

## Conclusions

Aromatase inhibitor treatment at follicle diameters of 12 mm or 18 mm or 24 h post ovulation were associated with a transient decrease in E_2_ concentrations and elevated circulating FSH and LH concentrations during the 4 days following treatment. The drop in E_2_ concentration did not suppress dominant follicle growth or early corpus luteum development. These results were unexpected and provide an impetus for additional studies to elucidate the roles of E_2_, LH and FSH in the regulation of follicle and luteal development in women. There is no reason to suspect other commercially available aromatase inhibitors (i.e., anastrozole) would have different effect than the observations made with letrozole on ovarian function.

## Abbreviations

AI: Aromatase inhibitors
BMI: Body mass index
E_2_: Estradiol
FSH: Follicle stimulating hormone
HAF: Hemorrhagic anovulatory follicle
LH: Luteinizing hormone
LUF: Luteinized unruptured follicle
OV: Ovulation
P_4_: Progesterone
WHIRL: Women’s Health Imaging Research Laboratory
